# Role of Oxidative Stress in HIV-1-Associated Neurocognitive Disorder and Protection by Gene Delivery of Antioxidant Enzymes

**DOI:** 10.3390/antiox3040770

**Published:** 2014-11-18

**Authors:** Jean-Pierre Louboutin, David Strayer

**Affiliations:** Department of Pathology, Anatomy and Cell Biology, Thomas Jefferson University, 1020 Locust street, Philadelphia, PA 19107, USA; E-Mail: David.Strayer@jefferson.edu

**Keywords:** HIV-1, gene therapy, dementia, oxidative stress, neuroinflammation, antioxidant enzymes

## Abstract

HIV encephalopathy covers a range of HIV-1-related brain dysfunction. In the Central Nervous System (CNS), it is largely impervious to Highly Active AntiRetroviral Therapy (HAART). As survival with chronic HIV-1 infection improves, the number of people harboring the virus in their CNS increases. Neurodegenerative and neuroinflammatory changes may continue despite the use of HAART. Neurons themselves are rarely infected by HIV-1, but HIV-1 infects resident microglia, periventricular macrophages, leading to increased production of cytokines and to release of HIV-1 proteins, the most likely neurotoxins, among which are the envelope glycoprotein gp120 and HIV-1 *trans*-acting protein Tat. Gp120 and Tat induce oxidative stress in the brain, leading to neuronal apoptosis/death. We review here the role of oxidative stress in animal models of HIV-1 Associated Neurocognitive Disorder (HAND) and in patients with HAND. Different therapeutic approaches, including clinical trials, have been used to mitigate oxidative stress in HAND. We used SV40 vectors for gene delivery of antioxidant enzymes, Cu/Zn superoxide dismutase (SOD1), or glutathione peroxidase (GPx1) into the rat caudate putamen (CP). Intracerebral injection of SV (SOD1) or SV (GPx1) protects neurons from apoptosis caused by subsequent inoculation of gp120 and Tat at the same location. Vector administration into the lateral ventricle or cisterna magna protects from intra-CP gp120-induced neurotoxicity comparably to intra-CP vector administration. These models should provide a better understanding of the pathogenesis of HIV-1 in the brain as well as offer new therapeutic avenues.

## 1. Clinical Presentation of HIV-1-Associated Neurocognitive Disorder

HIV-1 enters the Central Nervous System (CNS) soon after it enters the body. There, it is largely impervious to highly active anti-retroviral therapeutic drugs (HAART). Human immunodeficiency virus (HIV-1) encephalopathy covers a range of HIV-related CNS dysfunction. Advances in the treatment of HIV-1 have dramatically improved survival rates over the past 10 years, but HIV-associated neurocognitive disorders (HAND) remain highly prevalent and continue to represent a significant public health problem, partly because HAART penetrate the CNS poorly. In early 1990s, the neurologic complications of HIV-1 infection were classified into two levels of disturbance: (1) HIV-associated dementia (HAD) with motor, behavioral/psychosocial, or combined features; and (2) minor cognitive motor disorder (MCMD). HAD was considered as the most common cause of dementia in adults under 40 [1] and was estimated to affect as many as 30% of patients with advanced AIDS [1], but has become less common since HAART was introduced [2]. This reduction probably reflects better control of HIV in the periphery, since antiretroviral drugs penetrate the CNS poorly. Before the introduction of HAART, most neuroAIDS patients showed subcortical dementia, with predominant basal ganglia involvement, manifesting as psychomotor slowing, Parkinsonism, behavioral abnormalities and cognitive difficulties [3]. MCMD described a less severe presentation of HIV-associated neurocognitive impairment that did not meet criteria for HAD.

More recently, in light of the changing epidemiology of HIV infection, the need to update and further structure the diagnostic criteria for HAND has been recognized [4]. There are several reasons for this update. First, the applicability of the old criteria appears limited in the present age of HAART. Prior to the advent of HAART, a diagnosis of HAD was associated strongly with high viral loads, low T-cell counts, and opportunistic infections. With HAART limiting viral severity, patients with HIV typically live longer with milder medical symptoms. Secondly, guidelines regarding possible neurocognitive impairment due to comorbid conditions with CNS effects (e.g., substance use disorders) were not precisely described in the previous diagnostic scheme. This limitation is particularly important in the era of HAART as those infected with HIV-1 live longer with a host of CNS risk factors, including substance abuse disorders (e.g., methamphetamine dependence), medical conditions associated with HAART treatment (e.g., hyperlipidemia) and comorbid infectious diseases (e.g., hepatitis C virus) [5].

Thus, the newly redefined criteria allow for three possible research diagnoses: (1) asymptomatic neurocognitive impairment (ANI); (2) HIV-associated mild neurocognitive disorder (MND); and (3) HAD. In this classification, the diagnosis of HAND must be determined by assessing at least five areas of neurocognitive functioning known to be affected by HIV infection (e.g., attention/working memory, executive functions, speed of information processing, episodic memory, motor skills, language, and sensoriperception) [4].

However, if there are currently less cases of HAD as survival with chronic HIV-1 infection improves, the number of people harboring the virus in their CNS increases, leading to new HIV-1-related neurological manifestations. The prevalence of HAND therefore continues to rise, and less fulminant forms of HAND have become more common than their more severe predecessors [4,5]. HAND remains a significant independent risk factor for AIDS mortality [2,4,5,6,7,8,9]. Incident cases of HAND are accelerating fastest among drug users, ethnic minorities, and women [6,7,8,9]. The number of HIV-infected individuals over 50 years of age is rapidly growing, including patients taking HAART [9]. It has been suggested that in 10 years, 50% of AIDS patients in the United States will be over the age of 50. Moreover, it is becoming clear that the brain is an important reservoir for the virus, and that neurodegenerative and neuroinflammatory changes may continue despite HAART [8].

## 2. Neuropathogenesis of HAND

### 2.1. Role of HIV-1 Proteins in Neuronal Damage

The principal manifestations of central nervous system in HIV infection result from neuronal injury and loss and from extensive damage to the dendritic and synaptic structures in the absence of neuronal loss. Neurons themselves are rarely infected by HIV-1, and neuronal damage is felt to be mainly indirect. In fact, the pathogenesis of HAND largely reflects the neurotoxicity of HIV-1 proteins [10]. HIV-1 infects resident microglia, periventricular macrophages and some astrocytes [11], leading to increased production of cytokines and to release of HIV-1 proteins, the most likely neurotoxins, among which are the envelope (Env) proteins gp120 and gp41 and the nonstructural proteins Nef, Rev, Vpr and Tat [9,12,13].

#### 2.1.1. Trans-Acting Protein Tat

The HIV-1 *trans*-acting protein Tat, an essential protein for viral replication, is a key mediator of neurotoxicity. Brain areas that are particularly susceptible to Tat toxicity include the CA3 region and the dentate gyrus of the hippocampus and the striatum. Tat is internalized by neurons primarily through lipoprotein related protein receptor (LRP) and by activation of *N*-methyl-d-aspartate (NMDA) receptor [14,15]. It also interacts with several cell membrane receptors, including integrins, VEGF receptor in endothelial cells and possibly CXCR4 [16].

Tat can directly depolarize neuron membranes, independently of Na^+^ flux [17] and may potentiate glutamate- and *N*-methyl-d-aspartate (NMDA)-triggered calcium fluxes and neurotoxicity [17]. It promotes excitotoxic neuron apoptosis [18,19] by activating endoplasmic reticulum pathways to release intracellular calcium ((Ca^2+^)i). Consequent dysregulation of calcium homeostasis [18,20,21,22] leads to mitochondrial calcium uptake, caspase activation and, finally, neuronal death. Tat also increases levels of lipid peroxidation [23] by generating the reactive oxygen species (ROS) superoxide (O_2_^−^) and hydrogen peroxide (H_2_O_2_). It activates inducible nitric oxide synthase (iNOS) to produce nitric oxide (NO), which binds superoxide anion to form the highly reactive peroxynitrite (ONOO) [24].

Tat neurotoxicity has been reported in cultured cells*,* but fewer studies have demonstrated its neurotoxic properties *in vivo* [25,26,27,28,29]. Despite evidence that Tat has been detected in the striatum of patients with HIV encephalitis [30,31], it is difficult to know the exact levels of Tat generated. Although mRNA for Tat was detected by RT-PCR in brain extracts from half [31] or more [30] of patients with HIV encephalitis, protein levels could not be measured by ELISA [31]. There are important differences between the situation in the striatum of patients with AIDS and models where Tat is directly injected into the CP. In these models, Tat is localized initially in extracellular space following intra-CP injection, then it is internalized in neurons and in some microglial cells, while the sites of production are focal in patients with HAND (*i.e.*, microglial cells and infected macrophages). Direct injection of Tat results in acute injury, while production of Tat is more protracted in the brain of patients with HAND. The lesions of HAND reflect chronic injury caused by ongoing production of Tat, as well as other susbstances, by HIV-1-infected cells.

#### 2.1.2. Envelope Glycoprotein gp120

The HIV-1 *env* gene codes for gp120 which is cleaved into two major envelope glycoproteins, gp120 and gp41. Soluble gp120 can induce apoptosis in a wide variety of cells including lymphocytes, cardiomyocytes and neurons [32,33]. HIV-1 gp120 may be directly neurotoxic at high concentrations [34]. Gp120-induced apoptosis has been demonstrated in studies in cortical cell cultures, in rat hippocampal slices and by intracerebral injections *in vivo* [35]. Gp120 binds neuron cell membrane co-receptors (CCR3, CCR5 and CXCR4) and elicits apoptosis, apparently via G-protein-coupled pathways [21,36]. Soluble gp120 also increases glial cell release of arachidonate, which impairs neuron and astrocyte reuptake of glutamate [37], leading to prolonged activation of NMDA receptor with consequent disruption of cellular Ca^2+^ homeostasis [38]. This process involves generation of superoxide and peroxide species, with resultant oxidative stress, and leads to neuron cell death after mitochondrial permeabilization, cytochrome c release and activation of caspases and endonucleases [2].

#### 2.1.3. Other HIV-1 Proteins

The trans-membrane protein gp41 is elevated in patients with HAD. *In vitro*, gp41 can induce neuronal death in the nanomolar range, requires the presence of astrocytes, suggesting indirect mechanisms, involving iNOS, NO formation, depletion of glutathione and disruption of mitochondrial function [39,40].

Other HIV-1 proteins (Vpr, Nef, Rev) are also involved in HAND neuropathogenesis. HIV-1 viral protein r (Vpr) is thought to be important for effective viral replication in the early stages of the infection. Vpr is present as a soluble protein within the blood serum and the CSF of patients infected with HIV-1 [41,42] and accumulates within these compartments to increasing concentrations as disease progresses toward the later stages of disease. As an extracellular protein, HIV-1 Vpr has been shown to negatively affect the survival of brain-resident cells, especially neurons and astrocytes, which are the cell types most sensitive to local insult; they become dysfunctional and are gradually lost as patients infected with HIV-1 advance towards AIDS. Some studies have shown that Vpr can directly induce neuronal apoptosis [43], and that Vpr can deregulate calcium secretion in neural cells [44].

The non-structural protein Nef is required for the proper budding of virions from HIV-infected cells. *In vitro*, Nef can be lethal for astrocytes and neurons and can increase the expression of matrix metalloproteinases (MMPs) [45]. Abundant Nef expression has been shown in astrocytes of HIV-1-infected patients with neuronal damage [46].

The HIV-1 phosphoprotein Rev is involved in the nuclear export of unspliced viral mRNAs. Extracellular Rev has neurotoxic properties. These ones have been demonstrated in rodents by intracerebroventricular injection of a synthetic peptide spanning the basic region of Rev causing neuronal death [47].

### 2.2. HIV-1 Proteins and Astrocytes

Astrocytes have a role in HAND [48]. Several HIV-1 proteins can influence the role of astrocytes in HAND. Astroglial infection is characterized by an initial small burst in viral production followed by a state of persistent infection with the presence of multiple spliced short transcripts (encoding primarily the Nef protein), inefficient translation of structural proteins (gag and env), and almost undetectable levels of viral genomic transcripts [49]. Astrocytes could represent a reservoir for HIV-1. During late stage HIV-1 disease, an increased frequency of infiltrating monocytes and CD4+ T cells may deliver neurotoxic factors, such as chemokines and viral proteins (Tat, Vpr, gp120, and Nef) [50], which stimulates astroglia to secrete an elevated amount of glutamate, increasing the overall level of excitotoxicity. This sequence of events could play a significant role in astrocytic and neuronal dysregulation, leading to mild to severe neurocognitive impairment. Tat also induces NOS in human astroglia [51] and monocyte chemoattractant protein-1 (MCP-1) is induced in HIV-1 Tat-stimulated astrocytes [52]. Tat expression in astrocytes leads to astrocytes activation and neuronal death [53].

## 3. Oxidative Stress in HAND

### 3.1. Role of Oxidative Stress in HAND

Oxygen is vital for all living cells whether neuronal or not, but on the other hand it is potentially dangerous in excess. Oxygen has a role in glucose breakdown in mitochondria through oxidative phosphorylation and generates energy currency of cell, *i.e.*, ATP [54]. Under physiologic normal conditions, ROS, which include superoxide (O_2_^−^), hydrogen peroxide (H_2_O_2_) and hydroxyl radical (OH–), are generated at low levels and play important roles in signaling and metabolic pathways [55]. Oxidative stress arises due to the disturbances of the balance in pro-oxidant/antioxidant homeostasis that further causes the generation of ROS which are potentially toxic for neurons. Reactive oxygen and nitrogen species (ROS/RNS) change cellular responses through diverse mechanisms. Oxidative damage and the associated mitochondrial dysfunction may result in energy depletion, accumulation of cytotoxic mediators and cell death. There are several reasons why the brain is more susceptible to ROS. Glial cells require more oxygen and glucose consumption to generate continuous ATP pool *in vivo* for normal functioning of the brain as it is one of the busiest organs, making them more susceptible to oxygen overload, and thus to free radicals generation [54]. Neurons are particularly susceptible to ROS because of their biochemical composition: brain contains high levels of fatty acids, which are particularly susceptible to peroxidation and oxidative modification. Double bonds of unsaturated fatty acids are hot spots for attack by free radicals that initiate cascade to damage neighboring unsaturated fatty acids [56]. Membrane lipids can undergo oxidation, producing cytotoxic lipid peroxidation products like malondialdehyde (MDA) and 4-hydroxynonenal (4-HNE). Finally, brain has less antioxidant activity compared to other tissues and has higher levels of iron in some areas [54].

Organisms respond to oxidative injury by orchestrating a stress response to prevent further damage. An increase in the intracellular levels of antioxidant agents, and at the same time the removal of already damaged components, are both part of the oxidative stress response. ROS levels are controlled by endogenous antioxidants such as superoxide dismutases (SOD), glutathione peroxidase (GPx1), glutathione and catalase. The tripeptide glutathione (γ-l-glutamyl-l-cysteinylglycine, GSH) is the key low molecular thiol antioxidant involved in the defense of brain cells against oxidative stress. A decrease in GSH levels has been connected to physiological processes such as aging and neurological disorders like Alzheimer’s disease (AD), epilepsy, and Parkinson’s disease [57]. Although GSH is the primary molecule involved in detoxification of ROS in the body, antioxidant enzymes like GPx1, are also known to play a role in this process [58]. During detoxification of peroxides, the enzyme GPx1 converts GSH to GSSG (glutathione disulphide).

Interaction of ROS with other tissue components produces a variety of other radicals: following activation of iNOS, NO can bind superoxide anion to form the highly reactive peroxynitrite [24]. The latter may attack lipids, proteins and DNA, to enhance oxidant-related injury. Mitochondria are the primary source of ROS involved in many brain tissue injuries (*i.e.*, hypoxia, excitotoxicity). Once generated, mitochondrial ROS influence the release of cytochrome c and other apoptotic proteins from the mitochondria into the neuronal cytosol, which leads to apoptosis [55]. For example, once released into the cytosol, cytochrome c forms a complex referred to as an apoptosome with procaspase-9, apoptotic protease activating factor 1 (APAF-1) and dATP. The formation of the apoptosome activates caspase-9 which then cleaves other procaspases. The activation of caspase-3 by this process, among other effectors, has multiple effects including proteolysis of an inhibitor of the caspase-activated DNase [59]. Thus, a link between oxidative stress and activation of some caspases seems highly probable.

Abnormalities in oxidative metabolism have been reported in many nervous system diseases. These include neurodegenerative diseases (Parkinson’s disease, AD, Huntington’s disease, amyotrophic lateral sclerosis and cerebellar degeneration) [60,61,62,63], vascular diseases (ischemia-reperfusion) [64] or toxic reactions (chronic alcoholism) [65], as well as aging [66].

Oxidative stress, accumulation of protein aggregates, impaired mitochondrial function, and autophagic stress are common in many pathologies, including neurodegenerative diseases. The highly abundant mitochondria in brain cells are a major site of generation and action of ROS/RNS. Lipid peroxidation is a consistent feature of neurodegenerative diseases and biologically active reactive lipid species, such as HNE, accumulates in brains individuals with Parkinson’s disease, AD, and HIV-1-associated neurocognitive disorder. Mechanisms of protein oxidation are NO-dependent, through generation of ONOO−, or *S*-nitrosylation. These changes have been reported in a broad range of pathologies, including Parkinson’s disease, which is associated with both nitrated α-synuclein and *S*-nitrosated parkin. Likewise, ONOO−-dependent modifications of proteins are widespread in brains of individuals with AD. The cross-talk between autophagy, oxidative stress and mitochondrial dysfunction is not well understood, particularly in neurodegenerative diseases.

Oxidative stress plays a role in the development of HAND as well [67,68]. Oxidative stress in HIV-1 dementia has been documented by analyses of brain tissue, including increased levels of lipid peroxidation product (*i.e.*, HNE) and the presence of oxidized proteins. Serum levels of GSH and GPx1 are decreased in HIV-1 patients while MDA levels are increased [58]. A characteristic of patients infected with HIV-1 in late stage disease is diffuse intracellular oxidation in the form of decreased availability of GSH, the main cellular antioxidant and redox buffer, and augmented lipid oxidation, which triggers a cascade of downstream signaling events.

Membrane-associated oxidative stress correlates with HIV-1 dementia pathogenesis and cognitive impairment [9]. HNE-positive neurons have been demonstrated in the brains of patients with HIV-1 encephalitis [23,68,69]. In the case of HIV-1 infection, Tat and gp120 can elicit such oxidative stress [9,70]. Such oxidative stress can induce apoptosis in cultured neurons [71]. It can also damage neurons and cause cognitive dysfunction *in vivo* [72]. Tat and gp120 induce ceramide production in cultured neurons by triggering sphingomyelinase activity via a mechanism that involves induction of oxidative stress by CXCR4 activation [9]. Oxidative stress can play a role in HAND in other ways. Circulating toxins in the CSF, derived from HIV-1-infected cells, may damage mitochondria, leading to release of cytochrome c and then to a cascade of events leading to apoptosis [67,68]. HIV-1 gp120 and Tat can cause free radical production, possibly as part of the signal-transduction pathways they activate [9,70].

It is still unclear whether oxidative stress is the primary initiating event associated with neurodegeneration. However, a growing body of evidence implicates it as being involved in at least the propagation of cellular injury that leads to neuron death. Earlier reports support the hypothesis that oxidative modifications of macromolecular cell components (lipids, proteins and nucleic acids) may be an early step in the mechanism of Tat and gp120 neurotoxicity [27,28].

### 3.2. Oxidative Stress Associated with Tat

Tat-induced protein oxidation is well documented [27,28] and its effects on lipid peroxidation have also been reported. We recently demonstrated that Tat activates multiple signaling pathways. In one of these, Tat-induced superoxide acts as an intermediate, while the other utilizes peroxide as a signal transducer [70].

Tat-mediated neurotoxicity may be associated with increased oxidative modifications of proteins [27]. For example, increased protein carbonyl formation, a well-known marker of protein oxidative damage, occurred early after Tat injection and coincided with the earliest changes in the amount of degenerating striatal neurons [28]. When the number of degenerating neurons reaches its peak 1 day after Tat administration, protein oxidation in striatal extracts decreased back to control levels, probably because oxidized proteins are prone to proteolytic degradation [28]. There was a later increase in protein carbonyl levels 7 days after Tat injection, possibly caused by Tat-induced compromise astrocytic functions [28]. However, astrocytosis and associated changes in protein oxidation were not sufficient to cause an additional neuronal cell death [28]. Tat increase in levels of protein oxidation may result from Tat-mediated increase of the production of prooxidants, as Tat can trigger the production of inflammatory products, which, in turn, may cause an excess of ROS [28,73].

Tat can also mediate neurotoxicity through lipid peroxidation. There are few reports concerning MDA levels in the brain after Tat injection, and there are no data concerning late time points after inoculation. In one study, repeated intravenous injection of 50 ng Tat during 5 days decreases brain levels of GSH and GPx1 and increases levels of MDA [57]. Pretreatment of the animals with thiol antioxidant *N*-acetylcysteine amide (NACA) increased the GSH levels significantly, indicating that the antioxidant NACA was able to partially abrogate oxidative stress induced damage in these animals. A significant decrease in the activity of GPx1 was observed in animals treated with Tat, as compared to the controls, indicating that the overwhelming oxidative stress induced by these toxins depletes the antioxidant enzyme in the brain. Animals pretreated with NACA had GPx-1 levels similar to that of the control.

In one study, we injected Tat into the striatum. We observed elevated MDA levels persisting one week after Tat administration [74]. The sustained MDA levels might be due to the longterm neuroinflammation, because it is known that increased production of inflammatory products induced by Tat may cause an excess formation of ROS [28]. However, persisting elevated MDA levels were not associated with continuing apoptosis. These data resemble to the ones observed when an increase in protein carbonyl levels seen 7 days after Tat injection was not accompanied by neuron death [28].

Tat is also known to trigger an increased production of inflammatory products, which in turn, may cause an excess formation of ROS [73,75]. Tat may induce superoxide and nitrite release in a microglial cell line [73]. An exposure of macrophages and astrocytes to Tat for few minutes *in vitro* is sufficient for sustained release of cytokines for several hours [75]. Thus, Tat might promote oxidative stress and its consequences (*i.e.*, neuron death) through activation of proinflammatory responses.

Numerous neurotoxic effects of Tat injection can be transient and can be observed only at early time points after its administration. Tat can decrease levels of GSH available to relieve oxidant stress [57]. The presence of Tat in the striatum is short-lived after its injection. It is thus possible that after degradation of Tat, the levels of GSH are restored to normal levels. GSH could then protect neurons against ROS directly and indirectly, and could bind to lipid peroxidation products such as HNE, thereby providing neuroprotection [58]. Tat can also induce a lipid imbalance in neurons, resulting in an overproduction of sphingomyelin and ceramide, followed by increased levels of HNE [58]. Once Tat is degraded, the levels of ceramide and sphingomyelin return to control levels, limiting cellular dysfunction and death due to lipid imbalance. Tat can also trigger the expression of iNOS, leading to the overproduction of NO, which can react with superoxide anion to form peroxynitrite, a neurotoxic compound. NO can increase glutamate release from astrocytes, enhancing NMDA excitotoxicity [58]. If NO production can be increased shortly after Tat treatment, NO levels would decrease once Tat is degraded. Thus, if persisting increased MDA levels are observed one week after injection (possibly linked to continued neuroinflammation), they might not be enough, by themselves, to induce neuronal death at that time, because: (1) there is no direct interaction of Tat with neurons and no Tat-mediated dysregulation of calcium homeostasis one week after the injection; (2) some protective mechanisms (*i.e.*, GSH) are probably restored at that time; (3) neurotoxic compounds like NO and peroxynitrite are probably not present at that time.

Oxidative stress is intimately linked with an integrated series of cellular phenomena, which all seem to contribute to neuronal death. Interaction between these various components is not necessarily a cascade but might be a cycle of events, of which oxidative stress is a major component [76]. Consequently, if one of the events, besides oxidative stress, is missing, neuronal death might be limited or not occur. Inhibition of oxidative stress therapeutically might act to 'break the cycle' of cell death. It might also suggest that a direct interaction of Tat with neurons is necessary for inducing early neuron death. However, it is difficult to directly answer the question whether Tat directly induces oxidative stress in neurons or promotes it through activation of proinflammatory responses. It remains plausible that direct interactions of Tat with neurons play the role of a triggering mechanism in the process of the development of oxidative stress and neurodegeneration [28].

### 3.3. Gp120-Induced Oxidative Stress

It has been previously shown that HIV-1 gp120 can cause lipid peroxidation and production of hydroxynonenal esters [69], which can mediate oxidative stress-induced apoptosis of cultured neurons [77] and can damage neurons and cause cognitive dysfunction *in vivo* [72]. Recently, cytochrome P-450, in association with NADPH oxidase, has been implicated in gp120-induced oxidative stress and apoptosis in astrocytes [78].

We reported that direct injection of recombinant gp120 into the striatum can induce lipid peroxidation attested by the measurement of MDA and the production of HNE [79]. HNE was localized, not only in neurons, but also in endothelial cells and in astrocytes.

Experimental systems for studying the effects of gp120 and other HIV proteins on the brain have been limited to the acute effects of recombinant proteins *in vitro* or *in vivo*, or in chronic situation like simian immunodeficiency virus-infected monkeys. We described an experimental rodent model of ongoing gp120-induced neurotoxicity in which HIV-1 envelope gp120 is expressed in the brain using an SV40-derived gene delivery vector, SV(gp120) [80]. We previously demonstrated that SV40-derived vectors deliver long-term transgene expression to brain neurons and microglia, when administered by several different routes. rSV40s were employed in the current study because they transduce a wide range of cell types from humans and other mammals and deliver genes to cells in Go efficiently, including neurons, to achieve long-term transgene expression *in vitro* and *in vivo* [81,82]. Moreover, they do not elicit immune response [83]. These vectors transduce >95% of cultured human NT2-derived neurons, primary human neurons [84,85] and microglia [86] without detectable toxicity. When it is inoculated stereotaxically into the rat caudate putamen (CP), SV (gp120) caused a lesion in which neuron and other cell apoptosis continue for at least 12 weeks. Human immunodeficiency virus gp120 is expressed throughout this time, and some apoptotic cells are gp120 positive. MDA and HNE assays indicated that there was lipid peroxidation in these lesions. Similarly, protein oxidation was demonstrated by immunostaining for dinitrophenol (DNP) in brain cryosections, 1 week after injection of SV (gp120). Thus, *in vivo* inoculation of SV (gp120) into the rat CP causes ongoing oxidative stress and apoptosis in neurons and may therefore represent a useful animal model for studying the pathogenesis and treatment of HIV-1 envelope-related brain damage.

### 3.4. Oxidative Stress Associated with Vpr

Some recent studies have demonstrated the role of extracellular Vpr in impairing astrocytic metabolism by affecting the levels of the intracellular pools of both ATP and GSH, the main endogenous antioxidant molecule. Vpr-induced augmented production of ROS was related to an increase in the level of oxidized glutathione (GSSG) and a reduction in the overall GSH/GSSG ratio. This event was almost entirely suppressed by treatment with an anti-Vpr antibody or cotreatment with the antioxidant molecule *N*-acetyl-cysteine (NAC) [87].

## 4. Animal Models of HAND

There are no perfect models for HAND. Several animal systems have been used to study the pathogenesis of HIV-1-induced neurological disease. Many of them are based on other lentiviruses (*i.e.*, simian immunodeficiency virus infection of macaques, feline immunodeficiency virus infection of cats, Visna-Maedi virus infection in sheep) [88,89,90]. However, only small percentages of animals develop neurological manifestations in these models and the costs for using these species may be high. Transgenic expression of gp120 in mice has been studied [91], but the gp120 in that model is mainly expressed in astrocytes, whereas in humans HIV-1 chiefly infects microglial cells. Other models based on introduction of HIV-infected macrophages into the brains of SCID mice have been proposed, but they suffer from the fact of human macrophages delivered into a murine brain [92]. Some models of ongoing exposure to Tat have been developed. For example, GFAP-driven, doxycycline-inducible Tat transgenic mice have been useful for mechanistic studies of Tat contribution to HAND. However, the reported data concerning neuronal TUNEL positivity are still debated [93].

We [74,94,95] and others [26,96] have used model systems in which recombinant gp120, or Tat, proteins are directly injected into the striatum. The neurotoxicity of such recombinant proteins is highly reproducible and can be used as an interesting tool for testing novel therapeutic interventions. Administration of recombinant proteins is useful in understanding the effects of HIV-1 gene products, and so their individual contribution to the pathogenesis of HAND. However, HIV-1 infection of the brain is a chronic process, and its study would benefit from a model system allowing longer term exposure to HIV-1 gene product.

This is in part the reason why we developed experimental models of chronic HIV-1 neurotoxicity based on recombinant SV40 (rSV40) vector-modified expression of gp120 [80] or Tat [74] in the brain.

## 5. Antioxidant Therapeutic Approaches in HAND

As HIV-1 infection of the brain lasts the lifetime of affected individuals, and as eradication of CNS HIV-1 is currently not possible, control of the damage caused by the virus may represent a useful approach to treatment. This could entail limiting oxidative stress-related neurotoxicity.

### 5.1. Experimental Data

Antioxidant therapeutic options targeting oxidative stress can be artificially divided as targeting upstream and downstream pathways.

#### 5.1.1. Upstream Antioxidant Therapy

Upstream preventive treatment is based on prevention of free radical generation, regulation of neuronal protein interaction with redox metals (*i.e.*, Fe) and maintaining normal cellular metabolism.

Some components have been studied in models of HAND or can be useful in this context. Vitamin E can block the neurotoxicity induced by CSF of patients with HIV dementia [68]. Exposure to the soluble vitamin E analog Trolox prevents against neurodegenerative effects of recombinant HIV-1 Tat [97]. Some natural compounds have been tested in animal models of HAND. *Ginkgo biloba* extract EGb 761, through its antioxidant role, protects against HIV-1 Tat neurotoxicity [98].

Flavinoids are a group of compounds made by plants that have antioxidant and neuroprotective properties. This class of molecules has weak estrogen-receptor-binding properties and thus, do not have the side effects of estradiol. It has been described that diosgenin, a plant-derived estrogen present in yam and fenugreek can prevent neurotoxicity by HIV-1 proteins and by CSF from patients with HIV dementia [68]. Other interesting molecules have not been tested in HAND yet; they include resveratrol, found in grape skins, red wine, and peanuts, as well as genistein and quercetin, found in soybeans. Polyphenols are a group of compounds with antioxidant properties. Among them, curcumin can induce stress response-protective genes, such as heme oxygenase 1 (HO-1), and can protect against heavy-metal insult to the brain [58]. Selenium is a key molecule in GPx1 metabolism. Some HIV-infected patients have low levels of selenium. Because selenium supplementation increases GPx1 activity, it might be beneficial in these patients. *N*-acetyl-l-cysteine (NAC) is a nutritional supplement precursor in the formation of the antioxidant glutathione in the body and its sulfhydryl group confers antioxidant effects and is able to reduce free radicals. NAC injected i.p. into rodents increases glutathione levels in the brain and protects the CNS against the damaging effects of hydroxyl radicals and lipid peroxidation product acrolein [99]. *N*-acetylcysteine amide (NACA), a modified form *N*-acetyl-l-cysteine (NAC), where the carboxyl group has been replaced by an amide group, has been found to be more effective in neurotoxic cases because of its ability to permeate cell membranes and the blood-brain barrier (BBB). Treatment of animals injected intravenously with gp120, Tat and methamphetamine METH by NACA significantly rescued the animals from oxidative stress. Further, NACA-treated animals had significantly less BBB permeability as compared to the group treated with gp120 + Tat + METH alone, indicating that NACA can protect the BBB from oxidative stress-induced damage in gp120, Tat and METH exposed animals [57].

#### 5.1.2. Downstream Antioxidant Therapy

The therapeutic coverage of post oxidative stress events can be done by downstream antioxidant therapy. Non-steroidal anti-inflammatory drugs (NSAIDS) limit the infiltration of macrophages and can reduce the inflammatory cascade induced by oxidative stress. CPI-1189, a nitrone related compound, is supposed to regulate the pro-inflammatory cytokine cascade of genes in primary glial cells [54]. Minocycline is a tetracycline-derived compound that demonstrated neuroprotective profile in several models of neurodegeneration. The molecule has significant anti-inflammatory actions and can easily cross the BBB. *In vitro* data show that minocycline protected mixed neuronal cultures in an oxidative stress assay and has effective antioxidant properties with radical-scavenging potency similar to that of vitamin E [100]. Furthermore, minocycline treatment suppressed viral load in the brain, decreased the expression of CNS inflammatory markers and reduced the severity of encephalitis in a SIV model of HIV dementia [101]. A chemical moiety that resembles vitamin E in its chemical structure is the female sex hormone estrogen (estradiol) that contains a phenolic free radical scavenging site and acts as an antioxidant. It actually has the capability to prevent an upstream neurodegeneration and downstream the oxidative overload [54]. Estrogen replacement may result in improvement of cognitive function in several neurodegenerative disorders and conversely estrogen deficiency has been considered as a risk factor in some of them. Estradiol can protect against the neurotoxic effects of HIV-1 proteins in human neuronal cultures, probably by protecting the neuronal mitochondria in a receptor-independent manner [68]. However, estradiol has well known side effects in women (potential risk of developing breast or uterine cancer), and cannot be used in men or children because of feminizing effects [58]. It has been shown that several novel antioxidants (ebselen, diosgenin) can protect *in vitro* against neurotoxicity induced by CSF from patients with HV dementia [68].

It is likely that neuroprotective therapies should benefit from multiple and combination approaches targeting different aspects and pathways of the oxidative-stress insult. For example, coupling a potent antioxidant with a compound that modifies downstream signaling pathways (*i.e.*, minocycline) could provide a synergistic neuroprotective effects, at lower doses (and thus with less toxicity) that each molecule could achieve alone. The combination of HAART with an antioxidant compound and a molecule involved in downstream antioxidant therapy could be a promising avenue in the treatment of HAND [58]. However, it should be reminded that one of the challenges in designing antioxidants to protect the CNS against ROS is the crossing of the BBB.

### 5.2. Clinical Trials

A few antioxidants have been tried in small prospective controlled studies in HAND. However, the findings have all been relatively disappointing so far. Selegiline (l-deprenyl), which mechanism of action is speculative, albeit it might decrease the production of ROS and serve as an anti-apoptotic factor, was used in 2 double-blind controlled studies in the pre-HAART era. The first trial involving patients with minor cognitive and motor dysfunction (MCMD) showed improvement in verbal learning and trends for improvement in recall [102,103]. The second study was a smaller study in patients with MCMD and HIV dementia and showed significant improvement in delayed recall. However, other tests were not improved. A slight improvement was noted in patients treated with OPC-14117, a lipophilic compound structurally similar to vitamin E that acts as an antioxidant by scavenging superoxide radicals [104]. CPI-1189, a lipophilic antioxidant that scavenges superoxide anion radicals and block the neurotoxicity of gp120 and TNF-α [105], showed no effect on neurocognition in patients with MCMD and HIV dementia [106].

### 5.3. Gene Delivery of Antioxidant Enzymes in HAND

#### 5.3.1. Introduction

In order to deliver potent antioxidant compounds to the brain, we used gene transfer of antioxidant enzymes. Gene transfer of antioxidant enzymes has been studied in numerous models of neurological disorders by using diverse viral vectors [107,108,109]. We used rSV40 vectors to deliver SV (SOD1) or SV (GPx1) carrying the antioxidant enzymes Cu/Zn superoxide dismutase (SOD1) or glutathione peroxidase (GPx1) respectively, into the rat caudate putamen (CP). The safety of SV (SOD1) and SV (GPx1) delivered intra-CP has been demonstrated in rats and in Rhesus macaques monkeys, and resulting transgene expression is very durable [110]. Transgene expression of antioxidant enzymes can also be achieved through intravenous injection [111].

Mitochondria are a major site of production of superoxide in normal cells and probably contribute to increased oxidative stress in numerous diseases. Overexpression of mitochondrial Mn^2+^-superoxide dismutase results in moderate reductions in infarction in temporary ischemia. Glutathione, the major water-soluble antioxidant, is localized in both the cytosol and the mitochondria. Mice overexpressing the cytosolic enzyme Cu^2+^Zn^2+^-superoxide dismutase develop smaller infarcts than wild-type ones, with a decrease in multiple events associated with mitochondrially mediated apoptosis, including the release of cytochrome c [112]. It is thus possible that cytosolic overexpression of antioxidant enzymes delivered by SV40-derived vectors can mitigate the apoptotic events linked to mitochondria.

#### 5.3.2. Effects of Gene Delivery of Antioxidant Enzymes on Oxidative Stress, Apoptosis and Neuronal Loss in Animal Models of HAND

We showed that prior administration of recombinant SV40 vectors carrying antioxidant enzymes SOD1 or GPx1 protected either from Tat-induced oxidative injury (*i.e.*, lipid peroxidation) caused by intra-CP injection of Tat, or against SV (gp120)-induced oxidative injury. This reduction in oxidative stress due to gene transfer of antioxidant enzymes was associated with a protection against gp120- and Tat-elicited apoptosis and neuronal loss. Both striatal (either in acute or chronic models of HAND) and dopaminergic neurons were protected against gp120-induced insult [80,94,95,113,114].

Vector administration into the lateral ventricle (LV) [110] or cisterna magna (CM) [115], particularly if preceded by intraperitoneal mannitol, protects from intra-CP gp120-induced neurotoxicity comparably to intra-CP vector administration.

Caspases are involved in apoptosis linked to HAND. Caspases are implicated in neuronal death in neurodegenerative and other Central Nervous System (CNS) diseases.

Intrinsic apoptosis pathway is required for fetal and postnatal brain development, but is downregulated through the suppression of the expression of one of its key mediator, caspase-3 [114]. During stroke and neurodegenerative diseases, some caspases are upregulated in the brain [113]. Cerebral ischemia triggers both the intrinsic and extrinsic pathways of apoptosis [55,59]. Mounting evidence suggests the involvement of caspases in the disease process associated with neurodegenerative diseases such AD [115] and amyotrophic lateral sclerosis (ALS) [114].

The involvement of caspases in HIV-1 neurotoxicity has been documented *in vitro* and *in vivo*. Higher levels of caspase-3 and caspase-6 have been shown in the brains of patients with HAD [12,116,117]. Both HIV-1 neurotoxins gp120 and Tat significantly increase caspase-3 activation in striatal neurons *in vitro*. However, gp120 acts in large part through the activation of caspase(s), while Tat-induced neurotoxicity is also accompanied by activating an alternative pathway involving endonuclease G [118]. Tat can induce both caspases 3/7 and 9 in hippocampal cell cultures [119]. Increased expression of caspase-3 has been shown in neurons following exposure to Tat [18,21,71,77,118] and to gp120 [120,121,122]. In HIV-1 transgenic mice, Tat induction increased the percentage of neurons expressing caspase-3 [95]. Caspase-3-positive cells were also observed in a model of protracted exposure to gp120, SV(gp120) [80].

We studied the effect of gp120 on different caspases (3, 6, 8, 9) expression. Caspases production increased in the rat CP 6 h after gp120 injection into the same structure. The expression of caspases peaked by 24 h. Caspases colocalized mainly with neurons. There was a relationship with the concentration of gp120 injected. Both initiator (caspases 8 and 9) and effector/executioner (caspases 3 and 6) were increased after gp120 injection. We showed that about 70% of caspase-8- and 9-positive cells were TUNEL-positive while about 60% of caspase-3- and 6-positive cells were TUNEL-positive one day after intra-CP injection of gp120 [123]. Not all caspases-positive cells undergo apoptosis, at least as assessed by the methods used here and/or at the time points we considered. It is also possible that apoptosis will occur in the remaining caspases-positive cells at later time points. Gp120-induced caspase-3 activity may also be causing nonlethal neuron injury. As previously noted [95], if cell death in response to caspase-3 depends on total enzyme activity within a cell, the caspase-3 activity detected may be below the threshold required to initiate neuron death. This is difficult to determine based on immunocytochemistry. It has also been shown that activated caspase-3 rapidly degrades itself [124].

A link between oxidative stress and activation of some caspases seems highly probable. Prior gene delivery of the antioxidant enzymes SOD1 or GPx1 into the CP before injecting gp120 results in reduced levels of gp120-induced caspases, recapitulating the effect of antioxidant enzymes on gp120-induced apoptosis observed by TUNEL. Thus, HIV-1 gp120 increased caspases expression in the CP. Prior antioxidant enzyme treatment mitigated production of these caspases, probably by reducing ROS levels. While the present study strongly implicates caspases 3, 6, 8 and 9, additional studies are needed to determine the relative contribution of the various caspases to neuronal demise in HAND.

#### 5.3.3. Gp120-Mediated Abnormalities of the Blood-Brain Barrier are Mitigated by Gene Transfer of Antioxidant Enzymes

ROS are important in the pathogenesis of HIV-induced CNS injury [125] and can be induced in brain endothelial cells by HIV-1 gp120 and Tat [126,127,128]. Although damage to the BBB has been documented in patients with HIV-related encephalopathy [129,130,131], the exact mechanism by which this injury occurs is still debated [15,132,133,134,135,136,137,138,139,140,141]. We used animal models of HAND to characterize abnormalities of the BBB in this context. Exposure to gp120, whether acute (by direct intra-CP injection) or chronic using SV (gp120), an experimental model of ongoing production of gp120 disrupted the BBB, and led to leakage of vascular contents into the area of gp120 exposure. Gp120 was directly toxic to brain endothelial cells and gp120-mediated BBB abnormalities were related to lesions of brain microvessels [142]. Abnormalities of the BBB may reflect the activity of proteolytic enzymes, particularly matrix metalloproteinases (MMPs). MMPs are a family of neutral proteases that are grouped according to their protein structures. MMP-2 and MMP-9 are considered gelatinases [143], and are enzymatically activated by the cleavage of precursor propeptides. These target laminin, a major BBB component, and attack the tight junctions between endothelial cells and BBB basal laminae. MMP-2 and MMP-9 were upregulated following intra-CP gp120-injection. Gp120 greatly diminished total CP content of laminin and tight junction proteins. ROS have been reported to activate MMPs. Injecting gp120 into the CP induced lipid peroxidation, assessed by increased MDA levels. One product of gp120-triggered lipid peroxidation, HNE, was immunolocalized to vascular endothelial cells. Moreover, gene transfer of antioxidant enzymes using recombinant SV (SOD1) and SV (GPx1) protected against gp120-induced BBB abnormalities. BBB injury has also been linked to NMDA, which upregulates the proform of MMP-9 and increases MMP-9 gelatinase activity [144]. Using the NMDA receptor (NMDAR-1) inhibitor, memantine, we observed partial protection from gp120-induced BBB injury [79].

MMPs are upregulated in different neurological diseases and models of CNS injury [145,146,147,148,149,150,151]. Various factors, such as ROS, NO, and proteases such as plasmin and stromelysin-1, are involved in MMP activation and upregulation in CNS injury [152,153,154]. MMPs have been reported in the cerebrospinal fluid of HIV-1 infected patients [155,156] as well as in models of HIV-1 encephalopathy [157,158,159,160]. In rapidly progressing simian immunodeficiency virus-infected monkeys, MMP-9 levels correlate with motor and cognitive deficits [161]. Moreover, cerebrospinal fluid levels of the urokinase-type plasminogen activator receptor, which plays an important role in degradation of extracellular matrix, and hence BBB injury, are elevated in patients with HIV dementia [162].

Relatively little is known about the respective roles of ROS and oxidative stress in the balance between MMPs and their endogenous tissue inhibitors (TIMPs). More than 20 MMPs and four TIMPs act together to control tightly temporally restricted, focal proteolysis of extracellular matrix (ECM) [163]. Once activated, MMPs are subject to inhibition by specific TIMPs that bind MMPs non-covalently [163]. Tissue destruction by MMPs is regulated by TIMPs and TIMPs prevent excessive MMP-related degradation of extracellular matrix components. The balance between MMPs and TIMPs is linked to ECM remodeling and imbalance between TIMPs and MMPs can lead to excessive degradation of matrix components as in rheumatoid arthritis. Tumor metastasis and angiogenesis may also reflect such imbalances.

In the myocardium, ROS activate MMPs, decrease TIMPs levels and collagen synthesis [164]. A relationship between oxidative damage, MMP production and BBB disruption has been found in some lesions of the striatum [154]. We studied the effect of gp120 on TIMP1- and TIMP-2 production. TIMP-1 and TIMP-2 levels increased 6 h after gp120 injection into rat CP. TIMP-1 and TIMP-2 colocalized mainly with neurons (92% and 95% respectively). By 24 h, expression of these protease inhibitors diverged, as TIMP-1 levels remained high but TIMP-2 subsided. Gene delivery of the antioxidant enzymes SOD1 or GPx1 into the CP before injecting gp120 there reduced levels of gp120-induced TIMP-1 and TIMP-2, recapitulating the effect of antioxidant enzymes on gp120-induced MMP-2 and MMP-9. A significant correlation was observed between MMP/TIMP upregulation and BBB leakiness. Thus, HIV-1 gp120 upregulated TIMP-1 and TIMP-2 in the CP. Prior antioxidant enzyme treatment mitigated production of these TIMPs, probably by reducing MMP expression. This might be explained by reduced ROS generation, either as effectors of damage or as signaling intermediates, or both, by antioxidant gene transfer with subsequent decrease in MMP expression. Moreover, there was a significant correlation between gp120-related BBB disturbances and MMP/TIMP upregulation. Following prior antioxidant gene delivery, a relationship was also seen between the reduction in Evans Blue (EB) extravasation and MMP-9/TIMP-1 decreased production [165].

Thus, MMPs and their inhibitors, TIMPs, are upregulated in response to oxidative stress produced in a rat model of HIV encephalopathy. In this setting, increase TIMPs may counterbalance the increase in MMPs. The centrality of ROS to this process is demonstrated by the fact that prior gene delivery of antioxidant enzymes mitigates production of TIMPs, possibly by reducing MMP expression. These results suggest that gp120-related oxidative stress induces MMP upregulation, potentially which triggers TIMP production

#### 5.3.4. SV40-Mediated Gene Delivery of Antioxidant Enzymes Reduces gp120-Induced Neuroinflammation

If neuron loss [26,98,113,114] and astrogliosis [26] have been described in animals receiving gp120 directly into their brains, a temporal relationship between neuronal degeneration, astrocytic reaction, proinflammatory cytokine production and microglial proliferation remained to be established. We challenged rat CPs with 100–500 ng HIV-1BaL gp120, with or without prior rSV40-delivered SOD1 or GPx1. CD11b-positive microglia were increased 1 day post-challenge; Iba-1- and ED1-positive cells peaked at 7 days and 14 days respectively. Astrocyte infiltration was maximal at 7–14 days. MIP-1α was produced immediately, mainly by neurons. ED1- and GFAP-positive cells correlated with neuron loss and gp120 dose. We also tested the effect of more chronic gp120 exposure on neuroinflammation using an experimental model of continuing gp120 exposure. SV (gp120), a recombinant SV40-derived gene transfer vector was inoculated into the rat CP, leading to chronic expression of gp120, ongoing apoptosis in microglia and neurons, and oxidative stress. Increase in microglia and astrocytes was seen following intra-CP SV (gp120) injection, suggesting that continuing gp120 production increased neuroinflammation. SV (SOD1) or SV (GPx1) significantly reduced MIP-1α and limited neuroinflammation following gp120 administration into the CP, as well as microglia and astrocytes proliferation after injection of SV (gp120) in the striatum. Thus, gp120-induced CNS injury, neuron loss and inflammation may be mitigated by antioxidant gene delivery [166]. Similar results were observed when we injected Tat in the CP instead of gp120 [73].

Free radical production may be accompanied by elevated expression of MIP-1 alpha contributing to microglial recruitment and delayed neuronal death in several models of CNS injury [153,167,168,169]. The radical scavengers, like vitamin E analogs may inhibit free radicals and MIP-1α production, and recruitment of microglia in the injured area [169]. Our findings extend the principle of antioxidant protection from neuroinflammation to HIV-related injury, and suggest that rSV40 antioxidant gene delivery may be therapeutically applicable in the case of ongoing injury and neuroinflammation such as HAND.

HIV-1 envelope gp120 induces neuroinflammation when injected in the rat CP. Gp120-induced neuroinflammation correlates with neuron loss. An increase in expression of MIP-1α may play a role in this phenomenon, as well as ROS, as evidenced by the protective effects of rSV40-delivered antioxidant enzymes. The participation of other chemokines/cytokines in gp120-induced lesions *in vivo* remains to be established. The modulation of the interaction between these chemokines/cytokines and their ligands needs to be investigated.

## 6. Conclusions

HIV-1-associated neurocognitive disorder (HAND) is an increasingly common, progressive disease characterized by neuronal loss and progressively deteriorating CNS function. HIV-1 gene products, particularly gp120 and Tat, elicit ROS that lead to oxidant injury, cause neuron apoptosis, as well as subsequent consequences (e.g., neuroinflammation, abnormalities of the BBB). Understanding of, and developing therapies for, HAND requires accessible models of the disease. We have devised experimental approaches to studying the acute and chronic effects of gp120 and Tat on the CNS. These approaches to gp120 and Tat administration may therefore represent useful animal models for studying the pathogenesis and treatment of HIV-1 gp120- and Tat-related damage. Gene delivery of antioxidant enzymes by recombinant SV40-derived vectors protects against gp120 and Tat-induced oxidative stress and neuronal apoptosis, opening new avenues for potential therapeutics of HAND.
